# MicroRNA Expression Profiles Identify Biomarker for Differentiating the Embolic Stroke from Thrombotic Stroke

**DOI:** 10.1155/2018/4514178

**Published:** 2018-12-06

**Authors:** Lai-Te Chen, Chen-Yang Jiang

**Affiliations:** ^1^Zhejiang University, School of Medicine, Hangzhou, Zhejiang Province, China; ^2^Sir Run Run Shaw Hospital, Zhejiang University, School of Medicine, Hangzhou, Zhejiang Province, China

## Abstract

In order to identify potential biomarkers that distinguish the embolic stroke (ES) from thrombotic stroke (TS), a profile of microRNA expression was analyzed. The GSE60319 expression profile was downloaded from the Gene Expression Omnibus (GEO) database. The GEO2R was applied to screen for differentially expressed microRNAs (DEmiRNAs) between the embolic stroke group and thrombotic stroke group. The miRWalk was utilized to predict the target genes of DEmiRNAs. Genes associated with embolic stroke were downloaded from the Comparative Toxicogenomics Database. Cross reference of target genes to disease related genes was conducted to construct the DEmiRNA-gene network. The protein-protein interaction (PPI) network of overlapping genes was evaluated by STRING, using the MCODE and CytoHubba plugin of Cytoscape to identify the modules and hub genes. The enrichment of Kyoto Encyclopedia of Genes and Genomes (KEGG) in modules was performed. There were 30 microRNAs in total identified as DEmiRNAs between embolic stroke and thrombotic stroke groups, of which 8 were upregulated and 22 were downregulated. Among these differentially expressed miRNAs, miR-15a-5p, miR-17-5p, miR-19b-3p, and miR-20a-5p were significantly associated with an ES to TS. Using the miRWalk 3.0 online tool, target genes regulated by DEmiRNAs were predicted. In addition, disease related genes were predicted and compared with target genes of DEmiRNAs. 166 overlapped genes regulated by miR-15a-5p, miR-17-5p, miR-19b-3p, and miR-20a-5p were identified, suggesting their association with diseases that contributed to ES, mainly including atrial fibrillation, mitral valve stenosis, myocardial infarction, and aortic dissection. Therefore, miR-15a-5p, miR-17-5p, miR-19b-3p, and miR-20a-5p were promising candidate biomarkers for differentiating an ES from TS. The PPI network demonstrated that miR-15a-5p, miR-17-5p, miR-19b-3p, and miR-20a-5p were associated with an ES by mainly regulating “CCND1, E2F2, E2F3, ITCH, UBE4A, UBE3C, RBL2, FBXO31, EIF2C4, and EIF2C1”. Furthermore, miR-15a-5p and miR-17-5p may function through “cell cycle, prostate cancer, and small cell lung cancer” while miR-19b-3p and miR-20a-5p function through “insulin resistance, hepatitis B, and viral carcinogenesis” and “vasopressin-regulated water reabsorption”, respectively. However, these results were approached in the manner of bioinformatics analysis; therefore, further verification is required.

## 1. Introduction

Stroke is a complicated disease with significant morbidity which inevitably creates a heavy burden in clinical care and medical expenses [1]. The diversity in etiologies of stroke requests specific treatment and preventive strategies accordingly. Antiplatelet therapy is applied to thrombotic stroke while anticoagulant is indicated for cardioembolism caused by atrial fibrillation [2]. Therefore, further investigations of distinguishing embolic stroke from thrombotic stroke are required.

A few studies have tried to investigate the miRNA expression in patients with stroke which revealed that upregulation of miRNA-145 and downregulation of miRNA-210 contributed in ischemic stroke [3]. Key factors in thrombotic stroke such as angiogenesis, lipid metabolism, and neointimal formation were found to be regulated by microRNAs [4]. However, the possible role miRNA plays in subtypes of stroke remains unclarified.

In order to identify the potential role of miRNAs in distinguishing embolic stroke from thrombotic stroke, the GSE60319 dataset was downloaded from the Gene Expression Omnibus (GEO) database. Differentially expressed miRNAs (DEmiRNAs) were identified between embolic stroke and thrombotic stroke groups. Target genes of DEmiRNAs were predicted and compared with disease associated genes. DEmiRNA-gene network and protein-protein interaction (PPI) network were constructed to identify potential biomarkers and major regulated genes. Kyoto Encyclopedia of Genes and Genomes (KEGG) enrichment analysis was performed with modules of PPI network to assess the possible pathways.

## 2. Materials and Methods

### 2.1. Data Acquisition

The genetic expression profile of GSE60319 was downloaded from the Gene Expression Omnibus (www.ncbi.nlm.nih.gov/geo/), including 20 thrombotic stroke samples and 20 embolic stroke samples.

### 2.2. Identification of Differentially Expressed MicroRNAs (DEmiRNAs)

Different expression of microRNAs between embolic stroke and thrombotic stroke samples was extracted using GEO2R (http://www.ncbi.nlm.nih.gov/geo/geo2r/) which is an online analytic tool for performing comparison on raw data with the limma R packages and GEO query [5]. The results of comparisons were downloaded in the format of txt, and P<0.05 and |log fold-change| >3.5 were set as the threshold for differentially expressed microRNAs.

### 2.3. Prediction of Target Genes of Differentially Expressed miRNAs

Target genes of differentially expressed miRNAs were predicted using the miRWalk (version 3.0) (http://mirwalk.umm.uni-heidelberg.de/) [6]. Predicted genes which fitted all three databases (TargetScan, miRDB, and miRTarBase) were considered as target genes.

### 2.4. Prediction of Disease Associated Genes

Diseases such as atrial fibrillation, mitral valve stenosis, aortic dissection, etc. were major contributors to embolic stroke. Genes of certain diseases were downloaded from the Comparative Toxicogenomics Database [7] as disease associated genes.

### 2.5. Construction of DEmiRNA-Gene Network

Cross reference of disease associated genes to target genes was conducted and subsequently constructed a regulatory network with DEmiRNAs using Cytoscape software (version 3.6.1) [8].

### 2.6. Protein-Protein Interaction (PPI) Network Construction, Significant Modules Screening, and Hub Genes Establishment

Interaction among proteins were assessed with STRING (STRING; 2017 release) in the standard of combined score >0.4 [9]. The visualization of PPI network was accomplished with Cytoscape software (version 3.6.1). Additionally, the Cytoscape plugin Molecular Complex Detection (MCODE; version 1.31) and the Cytoscape plugin CytoHubba (version 0.1) were adopted to identify the significant modules and the top ranked genes in the PPI network [10, 11].

### 2.7. KEGG Enrichment Analysis

Kyoto Encyclopedia of Genes and Genomes (KEGG) enrichment analysis was conducted with the Enrichr in the standard of combined score >10 [12].

## 3. Results

### 3.1. Identification of DEmiRNAs

**Figure 1:**
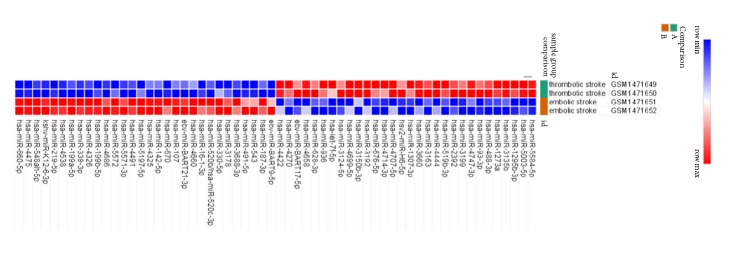
Heatmap of the top 30 DEmiRNAs. The dark green bar for thrombotic stroke group and the brown bar for embolic stroke group. Red for upregulated DEmiRNAs and blue for downregulated DEmiRNAs. DEmiRNAs: differentially expressed microRNAs.

**Figure 2:**
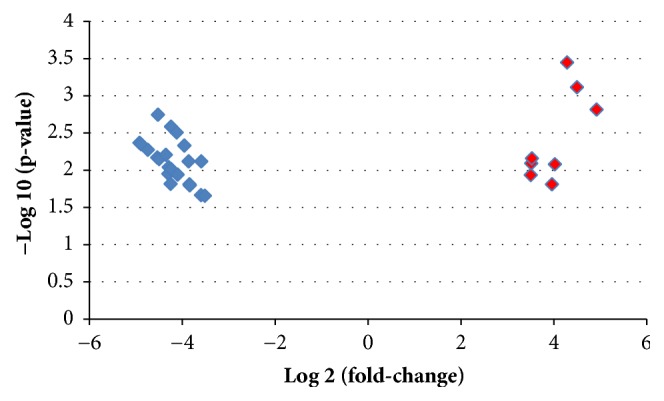
Volcano plot of DEmiRNAs. Diamond dots for DEmiRNAs with a |log2FC|>3.5. Blue diamond for downregulated DEmiRNAs. Red diamond for upregulated DEmiRNAs. DEmiRNAs: differentially expressed microRNAs.

By running expression profile of GSE60319 in NEO2R, amount of 30 differentially expressed miRNAs, including 8 upregulated DEmiRNAs and 22 downregulated DEmiRNAs, was identified and demonstrated with a heatmap (Figure 1) and a volcano plot (Figure 2).

### 3.2. Prediction of Target Genes

By using miRWalk online software, 170 target genes were predicted, all of which were downregulated.

### 3.3. Extraction of Disease Associated Genes and Construction of DEmiRNA-Gene Network

**Figure 3:**
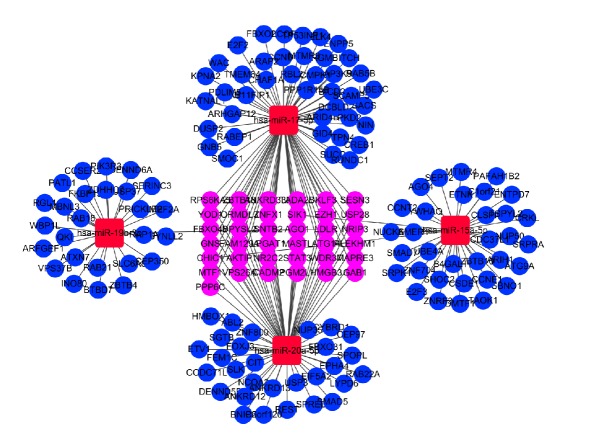
DEmiRNAs-gene network. Circular for genes, square for DEmiRNAs, blue for downregulated genes, red for upregulated DEmiRNAs, and purple for genes regulated by multiple DEmiRNAs. DEmiRNAs: differentially expressed microRNAs.

Disease associated genes were downloaded from the Comparative Toxicogenomics Database. Cross reference of disease associated genes to target genes was conducted. Using Cytoscape software (version 3.6.1), a regulatory network was constructed with overlapped genes and DEmiRNAs, in which 166 genes were regulated by 4 DEmiRNAs (miR-15a-5p, miR-17-5p, miR-19b-3p, and miR-20a-5p) (Figure 3).

Construction of protein-protein interaction (PPI) network, establishment of hub genes and major modules, and performance of KEGG analysis.

**Figure 4:**
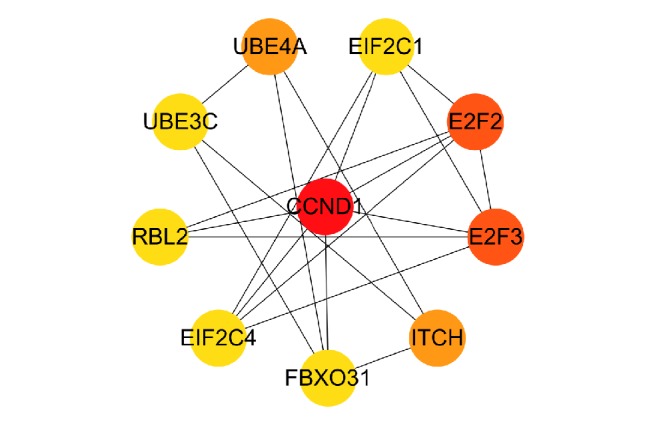
Top 10 hub genes. Color depth for ranking of hub genes.

The PPI network was constructed with 166 nodes and 116 edges. All genes were downregulated. With CytoHubba, ranking by MCC, hub genes were listed as follows: “CCND1, E2F2, E2F3, ITCH, UBE4A, UBE3C, RBL2, FBXO31, EIF2C4, and EIF2C1” (Figure 4).

**Figure 5:**
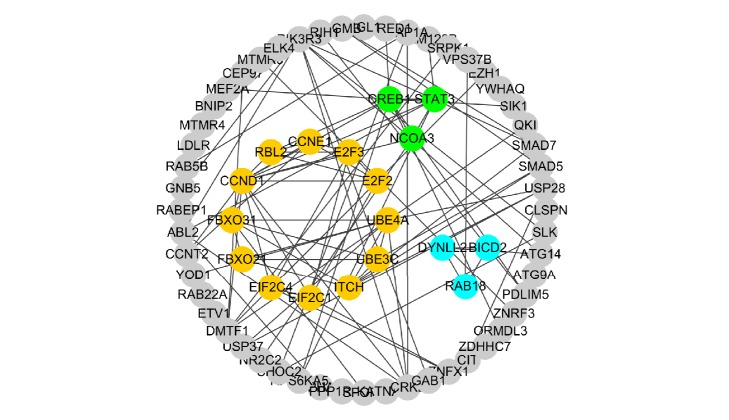
Modules of PPI. Circular for genes. Yellow for module 1, green for module 2, and cyan for module 3. PPI: protein-protein interaction.

**Figure 6:**
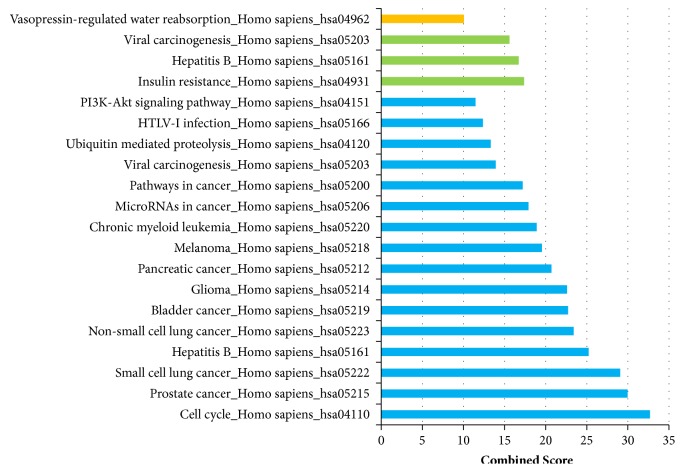
KEGG analysis of 3 modules. Light blue bars for pathways of module 1, green bars for pathways of module 2, and yellow bars for module 3. KEGG: Kyoto Encyclopedia of Genes and Genomes.

Using MCODE, 3 modules were identified, including module 1 with 12 genes which were significantly enriched in “cell cycle, prostate cancer, and small cell lung cancer”; module 2 with 3 genes which were mainly enriched in “insulin resistance, hepatitis B, and viral carcinogenesis”; module 3 with 3 genes which were mainly enriched in “vasopressin-regulated water reabsorption” (Figures 5 and 6).

## 4. Discussion

As demonstrated by published paper, anticoagulation is superior to antiplatelet in patients with embolic stroke caused by atrial fibrillation [13]. According to Chemtob RA, etc., the use of antiplatelet was tied to more incidences of intraoperative bleeding and transfusion requirement in patients with aortic dissection [14]. Thus it is important to identify the subtype of the ischemic stroke for therapeutic strategy on treatment and secondary prevention.

In the present study, by the criteria of P<0.05 and |log fold-change| >3.5, 30 differentially expressed miRNAs, including 8 upregulated DEmiRNAs and 22 downregulated DEmiRNAs, were revealed. Amount of 170 downregulated genes which simultaneously fitted 3 databases (TargetScan, miRDB, and miRTarBase) was pulled as target genes of DEmiRNAs. Genes associated with embolic stroke were predicted with the Comparative Toxicogenomics Database. Cross-reference the target genes to disease related genes; the overlapped ones regulated by miR-15a-5p, miR-17-5p, miR-19b-3p, and miR-20a-5p were used to construct a regulatory network. Possible mechanisms for involvement of these DEmiRNAs in subtypes of strokes were discussed in previous researches.

Fang L found that circulating miRNAs represented a novel biomarker of diffuse myocardial fibrosis. Overexpression of miR-15a-5p was found in hypertrophic cardiomyopathy (HCM) patients with diffuse myocardial fibrosis which increased ventricular stiffness, contributing to diastolic heart failure [15]. Heart failure, in turn, posed a higher risk for embolic stroke [16, 17]. As evidenced by published paper, elevated expression of serum miR-17-5p was associated with acute ischemic stroke [18]. Additionally, inhibition of microRNA-17-5p was found associated with decreased infarction areas and collagen fibers; inhibited apoptosis in cardiac tissues; promoted endothelial growth process, which improved cardiac function after acute myocardial infarction [19]. Hence, it was possible that upregulated microRNA-17-5p played a negative role in embolic stroke. Animal experiment showed that circulating level of miR-19b-3p was associated with cardiac dysfunction level during the development of diabetic cardiomyopathy [20], indicating possible involvement of miR-19b-3p in cardioembolic stroke. As a member of the miR-17 family, miR-20a-5p was well known of the biologic function in hepatic insulin resistance; however its function linked to skeletal muscle as a strength related miRNA emerged recently [21]. Bulent Vatan M reported altered expression of miR-20a-5p in patients with mitral chordae tendineae rupture, suggesting its potential target tissue of myocardium.

Ranked by MCC, the following genes were identified as hub genes in the present study: “CCND1, E2F2, E2F3, ITCH, UBE4A, UBE3C, RBL2, FBXO31, EIF2C4, and EIF2C1”, among which the roles of E2F2, E2F3, and EIF2C1 in neural diseases were established in previous studies. E2F2 and E2F3 were sensitized to neuronal cell loss after brain trauma [22, 23]. EIF2C1 was a central element of the microRNA translational silencing machinery which contributed in hypoxia-inducible factors related pathology such as stroke [24]. Research concentrated on prenatal diagnosis of congenital heart defects revealed that significant copy number variants of RBL2 were detected in fetuses with congenital heart defect [25]. Parallel study indicated that RBL2 in the duplicated region had been linked to heart defects and cardiac development [26]. Dysregulation of the CCND1 signaling pathway was responsible for the pathological process of ventricular remodeling [27], which subsequently turned into severe heart failure, conceiving high risk of embolic stroke. KEGG pathway analysis revealed that “PI3K-Akt signaling pathway” was significantly enriched for the miR-17-5p and miR-15a-5p. The role of PI3K-Akt signaling pathway acted in the brain injury caused by stroke was neuroprotective. Yu X suggested that the activation of PI3K-Akt signaling pathway served a positive role by attenuating oxygen-glucose deprivation injury [28].

## 5. Limitation

In this study, disease related genes were set as the genes which related to most etiologies of embolic stroke, such as atrial fibrillation, myocardial infarction, arterial dissection, and mitral valve stenosis. Paradoxical embolism caused by deep vein thrombosis was not addressed due to the relatively low incidence in embolic stroke [1]. Considering the prevalence of cardioembolism [29], it contained profound clinical significance even only cardioembolic stroke got distinguished from thrombotic stroke. Due to the lack of clinical samples with various etiologies of embolic stroke, experimental validations were not available. Thus, further evaluations in prospective trials are required to validate these biomarkers.

In conclusion, miR-15a-5p, miR-17-5p, miR-19b-3p, and miR-20a-5p are potential biomarkers for differentiating an embolic stroke from thrombotic stroke. The miR-17-5p may function by targeting hub genes like “CCND1, E2F2, UBE3C, RBL2, and ITCH”, while miR-15a-5p may contribute in identifying certain subtype of stroke by regulating E2F3, UBE4A, and EIF2C4. Both miR-15a-5p and miR-17-5p may function via PI3K-Akt signaling pathway. Targeting miRNAs of “miR-15a-5p, miR-17-5p, miR-19b-3p, and miR-20a-5p” would benefit the decision of clinical strategy by identifying the subtypes of stroke.

## Data Availability

“Data Availability” statement was included in the methodological part of the manuscript, labeled as “Data Acquisition”, content of which was listed as follows: The genetic expression profile of GSE60319 was downloaded from the Gene Expression Omnibus (www.ncbi.nlm.nih.gov/geo/); anything else in this manuscript was analysis based on the GSE60319.

## Abbreviations

GEO: Gene expression omnibus
DEmiRNAs: Differentially expressed microRNAs
PPI: Protein-protein interaction
GO: Gene ontology
KEGG: Kyoto Encyclopedia of Genes and Genomes
ES: Embolic stroke
TS: Thrombotic stroke
miRNAs: MicroRNAs.
